# Postprandial abdominal pain and weight loss as the initial presentation of abdominal neuroendocrine tumor recurrence

**DOI:** 10.1002/ccr3.9113

**Published:** 2024-06-23

**Authors:** Pitchaporn Yingchoncharoen, Kavya Bharathidasan, Jerapas Thongpiya, Diego Bernal, Mahmoud Abdelnabi

**Affiliations:** ^1^ Internal Medicine Department Texas Tech University Health Science Center Lubbock Texas USA

**Keywords:** mesenteric ischemia, neuroendocrine tumors, postprandial abdominal pain, somatostatin analogs, surgery

## Abstract

**Key Clinical Message:**

Neuroendocrine tumors, rare and slow‐growing, primarily affect the gastrointestinal tract, causing symptoms due to hormone secretion or mass effect. This case image described postprandial abdominal pain as an atypical initial presentation of abdominal neuroendocrine tumor recurrence in a middle‐aged male.

**Abstract:**

Neuroendocrine tumors are a group of rare, slow‐growing neoplasms, most commonly affecting the gastrointestinal tract. Clinical presentations include symptoms related to the mass or hypersecretion of hormones, such as flushing, diarrhea, or bronchoconstriction. Postprandial abdominal pain is most commonly related to chronic mesenteric ischemia from atherosclerotic changes but is rarely linked to external mass compression, including gastrointestinal tumors. Hereby, the authors highlight an uncommon presentation of NET, which is very challenging to diagnose and demands a high index of suspicion.

## 1 CASE PRESENTATION

**FIGURE 1:**
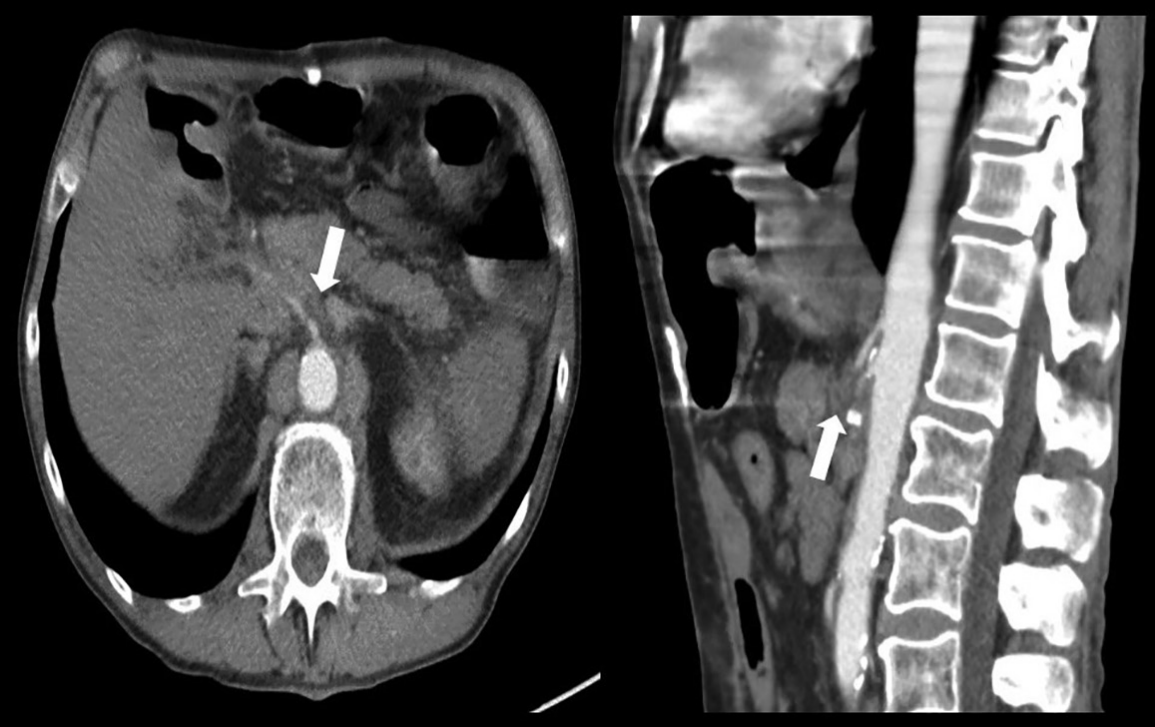
Computed tomography (CT) abdomen and pelvis with intravenous contrast (axial and sagittal planes) showing an ill‐defined soft tissue mass surrounding the celiac artery and superior mesenteric artery with dense calcification concerning for NET recurrence (arrow).

**FIGURE 2:**
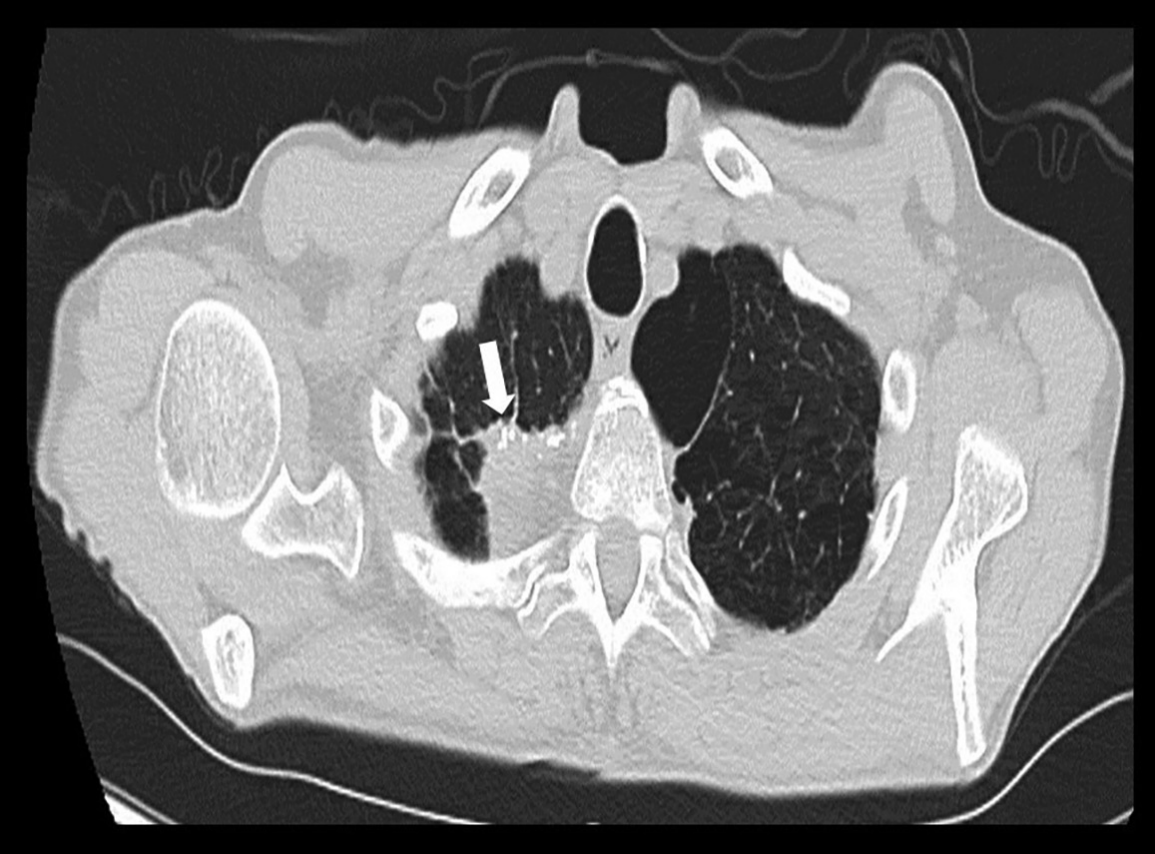
CT chest without contrast (axial plane) showing showed a 2.9 cm right apical mass concerning for metastatic disease (arrow).

A man in his 60s with a history of a small intestinal neuroendocrine tumor (NET) after status post resection complicated by anastomosis failure requiring extensive small intestinal resection and right hemicolectomy presented complaining of a 3‐month history of postprandial abdominal pain and significant weight loss of 25 pounds over the past 6 months. Clinical examination and laboratory workup were unremarkable, including normal gastrin except for markedly elevated chromogranin A. Computed tomography (CT) of the abdomen and pelvis with intravenous contrast (Figure 1) showed an ill‐defined soft tissue mass surrounding the celiac artery and superior mesenteric artery with dense calcification concerning for NET recurrence and retroperitoneal lymphadenopathy with lymph nodes measuring 2.3 cm. CT chest without contrast (Figure 2) showed a 2.9 cm right apical mass concerning for metastatic disease. Due to bleeding risk, endoscopic ultrasound was performed, showing two hypoechoic, well‐defined margins lymph nodes in the celiac region measuring 2.7 × 1.1 cm and 2.1 × 2 cm. Fine‐needle aspiration celiac lymph node biopsy revealed polymorphous lymphoid cells, most consistent with a reactive lymphoid population. Right upper lobe lung mass biopsy pathology showed alveolar lung parenchyma with fibrosis and chronic inflammation. He was counseled by a multidisciplinary team with oncology and surgery, and he opted to start chemotherapy instead of surgery due to complications and bleeding risks.

Neuroendocrine neoplasms constitute a group of tumors that can secrete functional hormones, resulting in diverse clinical manifestations, accounting for about 0.5% of all newly diagnosed malignancies.^1^ The annual age‐adjusted incidence of NETs has increased due to improved diagnostic modalities from 1.09 to 6.98 per 100,000 persons in 1973 and 2012, respectively. These tumors predominantly affect females in their 60s or older.^1^ Gastrointestinal and respiratory tracks (62%–67% and 22%–27%, respectively) are the most common primary sites of NETs. Characteristically, NETs grow slowly over time, with up to 22% of patients present with distant metastasis. Symptoms can be attributed to local mass effects or hormonal hypersecretion, including insulin, glucagon, gastrin, and vasoactive intestinal polypeptide in approximately 30% of cases.^2^ Gastroenteropancreatic NETs diagnosis can be challenging due to their varied clinical presentations, ranging from asymptomatic to nonspecific abdominal discomfort.^1^ Chronic mesenteric ischemia, an often‐overlooked cause of postprandial abdominal pain, is mainly caused by atherosclerotic occlusion of the mesenteric vessels, rarely linked to non‐atherosclerotic causes such as external compression.^3^ Surgical resection is the first‐line treatment for localized NETs, while palliative approaches are reserved for metastatic or bulky disease. Advances in treatment for advanced NETs include biological therapies such as somatostatin analogues for symptom control, targeted therapies like immune‐targeted therapies, peptide receptor radionuclide therapy, and liver‐targeted therapies for localized unresectable disease.^2^ Survival outcomes vary significantly based on the primary site, with rectal NENs showing a better prognosis (5‐year survival rate of 40%) compared to pancreatic NENs (5‐year survival rate of 22%).^1^

## AUTHOR CONTRIBUTIONS

**Pitchaporn Yingchoncharoen:** Writing – original draft. **Kavya Bharathidasan:** Validation; writing – original draft. **Jerapas Thongpiya:** Writing – original draft. **Diego Bernal:** Writing – original draft. **Mahmoud Abdelnabi:** Supervision; writing – review and editing.

## 2 FUNDING INFORMATION

None.

## CONFLICT OF INTEREST STATEMENT

None declared.

## CONSENT

Patient consent has been signed and collected in accordance with the journal's patient consent policy.

## Data Availability

All data underlying the results are available as part of the article, and no additional source data is required.

## References

[1] Dasari A , Shen C , Halperin D , et al. Trends in the incidence, prevalence, and survival outcomes in patients with neuroendocrine tumors in the United States. JAMA Oncol. 2017;3:1335‐1342. 28448665 10.1001/jamaoncol.2017.0589 PMC5824320

[2] Uri I , Grozinsky‐Glasberg S . Current treatment strategies for patients with advanced gastroenteropancreatic neuroendocrine tumors (GEP‐NETs). Clin Diabetes Endocrinol. 2018;4:16. 30009041 10.1186/s40842-018-0066-3 PMC6042326

[3] Biolato M , Gabrieli ML , Parente A , et al. Abdominal angina due to recurrence of cancer of the papilla of Vater: a case report. J Med Case Rep. 2009;3:9314. 20062743 10.1186/1752-1947-3-9314 PMC2803837

